# Balancing bleeding and valve thrombosis risk after transcatheter tricuspid valve replacement

**DOI:** 10.1093/ehjopen/oeae022

**Published:** 2024-03-23

**Authors:** Mathias Claeys, Geraldine Ong, Mark D Peterson, Sami M Alnasser, Neil P Fam

**Affiliations:** Structural Heart Program, St. Michael’s Hospital, Donnelly Wing, 30 Bond Street, Toronto, Ontario M5B 1W8, Canada; Structural Heart Program, St. Michael’s Hospital, Donnelly Wing, 30 Bond Street, Toronto, Ontario M5B 1W8, Canada; Structural Heart Program, St. Michael’s Hospital, Donnelly Wing, 30 Bond Street, Toronto, Ontario M5B 1W8, Canada; Structural Heart Program, St. Michael’s Hospital, Donnelly Wing, 30 Bond Street, Toronto, Ontario M5B 1W8, Canada; Structural Heart Program, St. Michael’s Hospital, Donnelly Wing, 30 Bond Street, Toronto, Ontario M5B 1W8, Canada

Transcatheter tricuspid valve replacement (TTVR) is a novel tool in the management of severe tricuspid regurgitation (TR). It is of particular interest for isolated functional TR where treatment often remains underwhelming due to the limited efficacy of medical therapy, poor outcomes with surgery, and the limitations of transcatheter valve repair. Early reports of TTVR have been encouraging and several valve systems are undergoing clinical evaluation. Optimal periprocedural management is still uncertain as evidence-based recommendations are lacking, resulting in wide variation between institutions. The goal of this letter is to share our experience regarding bleeding and thrombotic outcomes after TTVR and propose a framework for the periprocedural management of anticoagulation.

This is a retrospective analysis of patients undergoing compassionate use TTVR between June 2018 and December 2022 at St. Michael’s Hospital, Toronto, Canada. The study was approved by the local ethics committee. The data underlying this article will be shared on reasonable request to the corresponding author.

**Figure 1:**
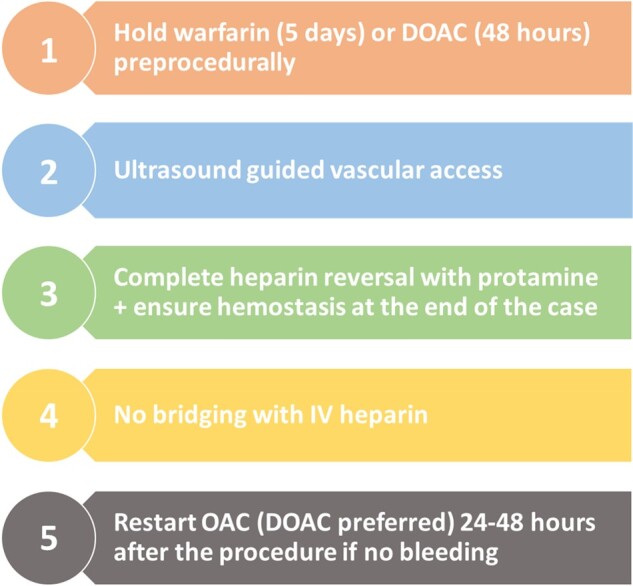
Proposed algorithm to balance bleeding and thrombosis after TTVR. OAC, oral anticoagulation; DOAC, direct oral anticoagulant.

We included 28 patients in this analysis. The median age was 78 years (interquartile range: 72–82 years), and 12 (43%) were women. Prior to the procedure, 25 patients (89%) were anticoagulated with either warfarin (*n* = 10), tinzaparin (*n* = 1) or a direct oral anticoagulant (DOAC, *n* = 14) for mechanical valves (*n* = 4), atrial fibrillation (*n* = 20) or prior thromboembolism (*n* = 1). TTVR devices included EVOQUE (Edwards Lifesciences, Irvine, CA), Cardiovalve (Cardiovalve Ltd, Or Yehuda, Israel), Topaz (TriCares, Paris, France), LuX-Valve Plus (Jenscare Scientific, Ningbo, China), and Navigate (NSCI, Lake Forest, CA). Patients received postprocedural anticoagulation with either warfarin (*n* = 12), tinzaparin (*n* = 1) or apixaban (*n* = 15). Twenty-six patients (93%) had successful device implantation. One patient had moderate paravalvular regurgitation, managed conservatively, and another patient who had severe PVL underwent successful surgical TVR. Mortality at 30-day follow-up was 4%, with one patient dying of hemothorax with subsequent septic shock during the index hospitalization. During follow-up (median 283 days, interquartile range 59–491), eight patients (29%) had a bleeding event, seven of them in the first three days and one on day 10. According to the VARC-2 bleeding scale, five bleeding events (18%) were minor (three access site, one epistaxis, and one gastrointestinal bleed) and three (11%) were major (one intrapulmonary haemorrhage, one access site, and one gastrointestinal bleed). Bleeding occurred predominantly while patients were receiving IV heparin bridging with warfarin (seven vs. one on DOAC and were proportionally higher in those receiving aspirin (5/11) vs. those without (3/17). Five patients (18%) required postprocedural blood transfusion. Access site bleeding occurred exclusively in patients without postprocedural heparin reversal with protamine. Valve thrombosis, defined as hypoattenuating leaflet thickening (HALT), reduced leaflet mobility or a sudden increase in valve gradient, was noted in three patients (11%) and occurred 4–21 days after the procedure. In two patients, valve thrombosis occurred after bleeding complications requiring transfusion and one patient had heparin-induced thrombocytopenia. Valve thrombosis was successfully managed with IV heparin or argatroban resulting in a return of normal valve function at follow-up. The high bleeding prevalence prompted a change in periprocedural management from a protocol of warfarin with IV heparin bridging plus aspirin towards a less aggressive approach (*Figure 1*). This was associated with a reduction in bleeding [6/12 pre vs. 2/16 post (50% vs. 13%), Fisher Exact *P* = 0.044], with only two minor bleeding events and without any clinical valve thrombosis.

Postprocedural bleeding is frequent after TTVR and reflects the heightened risk of anticoagulation in these frail patients with multiple comorbidities in addition to procedural characteristics (large bore access) and early experience. In our cohort, severe bleeding occurred in 11% of patients, compared to 27% observed in the TRISCEND study.^1^ Bleeding occurred almost entirely in patients on warfarin bridged with IV heparin and those without postprocedural heparin reversal. Valve thrombosis on the other hand was less common, resolved with intensive anticoagulation and generally occurred later compared to bleeding. Hence, as the bleeding risk outweighs the risk of valve thrombosis in the early postprocedural phase, our data seems to favour a less aggressive approach to anticoagulation (*Figure 1*). This includes ultrasound-guided vascular access, postprocedural heparin reversal with protamine, avoidance of periprocedural bridging (unless mechanical valves), or concomitant antiplatelet therapy, along with confirmation of adequate hemostasis prior to resuming therapeutic anticoagulation, preferably with DOAC (apixaban) 24–48 h after the procedure.

This study has several limitations, including its small sample size, single center experience, retrospective study design and the potential heterogeneity due to the inclusion of different valves. Although all patients had echo follow-up, CTs were not routinely performed and subclinical valve thrombosis/HALT can therefore not be excluded.

In conclusion, our results show bleeding is common after TTVR while valve thrombosis is less frequent and occurs later. TTVR patients might therefore benefit from a less aggressive approach to anticoagulation, with a focus on optimal hemostasis in the immediate postprocedural period, although this remains to be confirmed in larger, prospective trials.
